# Acute obstructive uropathy secondary to a rectus sheath haematoma in an anticoagulated patient

**DOI:** 10.1093/jscr/rjae745

**Published:** 2024-11-24

**Authors:** Stephanie M Babic, Anand Trivedi

**Affiliations:** Acute Surgical Unit, Fiona Stanley Hospital, 11 Robin Warren Drive, Murdoch, Western Australia, 6150, Australia; Acute Surgical Unit, Fiona Stanley Hospital, 11 Robin Warren Drive, Murdoch, Western Australia, 6150, Australia

**Keywords:** rectus sheath haematoma, obstructive uropathy, anticoagulation, ureteric stenting, extravasation

## Abstract

A rectus sheath haematoma (RSH) is a relatively rare cause of acute abdominal pain that is becoming more prevalent due to an increase in anticoagulant therapy. Of its associated complications, acute obstructive uropathy is exceedingly rare. This is a case of a 62-year-old lady who presented with abdominal pain caused by an RSH which then led to obstructive uropathy. She had undergone laparoscopic removal of a gastric band 6 days prior and due to a mechanical mitral valve, required ongoing anticoagulation. Initially, she had a contained RSH, but this subsequently decompressed into the extraperitoneal space, causing acute obstructive uropathy secondary to external compression. She was managed with ureteric stenting and her anticoagulation was appropriately modified throughout her admission. This case highlights that the challenging aspect of RSH management involves tailoring treatment to address individual patient factors and the location of the haematoma itself.

## Introduction

A rectus sheath haematoma (RSH) is a relatively rare but recognised cause of acute abdominal pain, involved in less than 2% of cases [1, 2]. However, recent studies note the rising prevalence of RSHs due to increasing anticoagulant use [3, 4]. This increase is demonstrated in Australia, which saw a one-third rise in oral anticoagulant prescriptions from 2009 to 2018 [5]. RSHs can be spontaneous or caused by iatrogenic or traumatic injury [3]. Typically, an RSH presents with abdominal pain, a palpable abdominal wall mass and haemoglobin drop, with or without haemodynamic instability [1]. Rarely, an RSH can cause obstructive uropathy or abdominal compartment syndrome [1, 6–9]. Recent studies have developed algorithms to assist with managing RSHs, but these have not been prospectively tested and do not account for complex patient factors [10, 11]. This is a case of a patient with an RSH causing acute obstructive uropathy that required ureteric stenting but was otherwise managed conservatively due to the patient’s complex medical history and the complexity of the haematoma itself.

## Case

A 62-year-old lady presented with left upper quadrant abdominal pain that had started suddenly the day prior and progressively increased in severity. She was 6 days post-elective laparoscopic removal of a gastric band (originally placed in 2007). The procedure was uncomplicated and she had been discharged two days prior with a haemoglobin of 95 g/L. She was on warfarin 7.5 mg daily for a metallic mitral valve inserted in 2013 for rheumatic heart disease. She was on bridging enoxaparin injections and had recommenced warfarin day one post-procedure. Otherwise, her medical history included atrial fibrillation, asthma and chronic obstructive pulmonary disease. She was independent, a non-smoker and had minimal alcohol intake.

**Figure 1:**
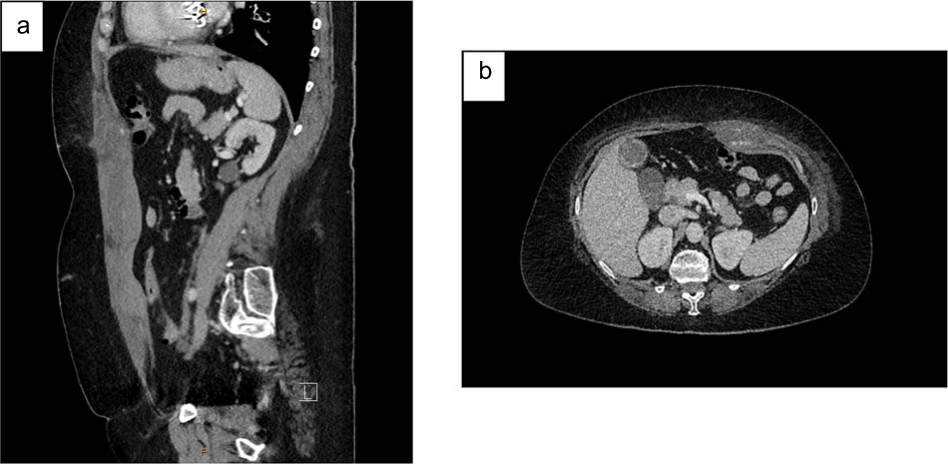
Contrast CT abdomen and pelvis (portal venous phase) demonstrating a left-sided RSH with active extravasation: (a) Sagittal view; (b) Axial view.

On examination, she was haemodynamically stable and afebrile. She had a soft abdomen, widespread abdominal bruising, and significant tenderness to the left of her umbilicus, without a discrete palpable mass. Urinalysis showed a trace of leucocytes but no blood or nitrites. Her blood tests showed haemoglobin 94 g/L, international normalised ratio 1.9, creatinine 63 μmol/L and estimated glomerular filtration rate (eGFR) >90 mL/min/1.73m^2^. A CT abdomen showed a left-sided RSH (62x38x131mm), with high-attenuating foci superiorly and inferiorly (Fig. 1). She was admitted to the Acute Surgical Unit and warfarin was withheld but therapeutic enoxaparin was continued on the advice of the Haematology team.

**Figure 2:**
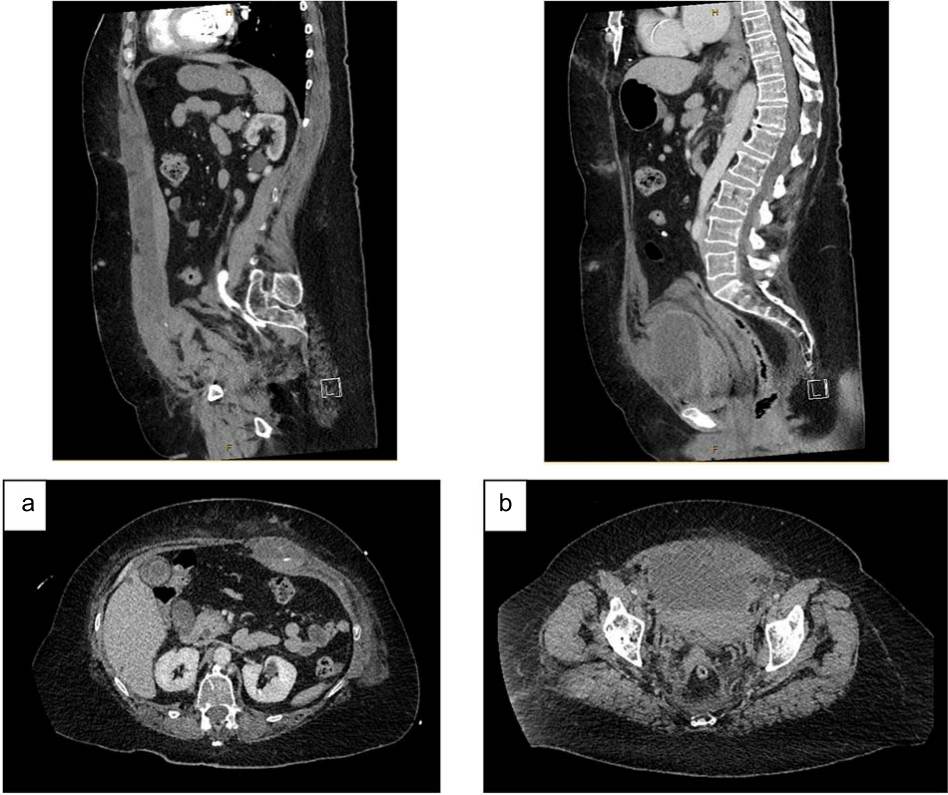
CT angiography (portal venous phase): (a) increased size of RSH and ongoing active extravasation – sagittal (top) and axial (bottom) views. (b) Extraperitoneal haematoma compressing and displacing urinary bladder – sagittal (top) and axial (bottom) views.

On Day 2, she became haemodynamically unstable with heart rate 150 bpm, systolic blood pressure 84 mmHg and a haemoglobin drop to 84 g/L. She was given oral Vitamin K and transfused packed red blood cells (PRBC) and fresh frozen plasma. CT angiography showed an increased RSH (61x36x301mm) with multiple foci of arterial enhancement, and a large pelvic extraperitoneal haematoma (119x81x121mm) displacing the urinary bladder posteriorly with active extravasation posterior to the left superior pubic ramus (Fig. 2). The Surgical team held extensive discussions with Interventional Radiology, Intensive Care, Haematology, Cardiology and Urology. The balance was in favour of ongoing conservative management due to (i) the thrombosis risk associated with discontinuing anticoagulation and (ii) the technical difficulty and risks of embolisation in an anticoagulated patient with multiple foci of arterial extravasation.

She was transferred to the Intensive Care Unit and required intermittent inotropic support and daily PRBC transfusions to maintain a haemoglobin target >80 g/L. On Day 4, she became anuric and her renal function deteriorated, showing creatinine 283 μmol/L and eGFR 15 mL/min/1.73m^2^. A non-contrast CT demonstrated a stable RSH but increased extraperitoneal pelvic haematoma (180x150x117mm) and bilateral hydronephrosis. The Urology team performed a rigid cystoscopy and bilateral ureteric stenting. Post-operatively, the consensus was to commence a heparin infusion and continue conservative management of the haematomas. Ultimately, the patient’s renal function recovered, and she remained stable and was discharged on Day 11 on therapeutic enoxaparin, with a plan to re-commence warfarin 2 weeks later.

## Discussion

The case presented deviates from the ‘typical’ RSH, as it caused significant acute obstructive uropathy and was overtly complex to manage due to both patient and pathology factors. Management options were limited due to the risk of withholding, let alone reversing, anticoagulation in a patient with a mechanical mitral valve.

Several recent studies have developed algorithms to guide RSH management. Contrella *et al.* identified clinical and radiological factors associated with failure of conservative management [10]. The authors suggest that embolisation should be performed for active extravasation on CT, haematoma volume ≥ 1300 ml, transfusion ≥ four units PRBCs, or haemoglobin drop ≥ 25 g/L. Similarly, Angeramo *et al.* developed an algorithm using Berna CT grade [12], patient age, haematoma volume, haemodynamic stability, haemoglobin drop, and haematocrit drop to decide on conservative or interventional management [11].

According to these proposed algorithms, the RSH in this case would ordinarily be managed with embolisation due to active extravasation, haemodynamic instability, haematoma size and transfusion requirement. However, these algorithms have not been prospectively tested, and even an optimal algorithm cannot account for complex patient factors. Sheth *et al*. [3] highlighted that most patients with an RSH are on anticoagulation for a variety of reasons, including cardiac valve replacement and the indication must be carefully considered. The anticoagulation management plan in this case was formulated by several teams, using a balanced approach to mitigate both bleeding and thrombosis risks.

Embolisation for RSHs is often technically successful. However, morbidity and mortality remain high, due to patient comorbidities and low physiological reserve [10]. In this case, embolisation was discussed at length between teams. However, multiple foci of active bleeding within the RSH and the extraperitoneal haematoma in a coagulopathic patient meant that embolisation was unlikely to be technically successful. Similarly, surgical management of the RSH was not considered a viable option.

## Conclusion

An RSH is a relatively rare cause of acute abdominal pain and for it to result in acute obstructive uropathy is rarer still. Several recent algorithms provide guidance on whether to manage an RSH conservatively or with intervention. However, individual patient factors and the haematoma location, morphology and complications must be considered. In this case, the patient required ureteric stenting but was otherwise managed conservatively, due to ongoing anticoagulation requirements and haematoma complexity. Close patient monitoring and multidisciplinary team decision-making was required to ensure success using this approach.
